# And yet, migration, population growth or mortality are not balanced among Cantabrian brown bear subpopulations. Reply to Blanco et al (2020)

**DOI:** 10.1371/journal.pone.0256432

**Published:** 2021-10-13

**Authors:** Eduardo Ferreira, Inês Gregório, Tânia Barros, Doriana Pando, Joaquín Morante, Ana Barbosa, Roberto Hartasánchez, Carlos Fonseca

**Affiliations:** 1 Department of Biology & CESAM, University of Aveiro, Campus Universitário de Santiago, Aveiro, Portugal; 2 Fondo para la Protección de los Animales Salvajes, Santo Adriano, Asturias, Spain; 3 ForestWISE—Collaborative Laboratory for Integrated Forest & Fire Management, Quinta de Prados, Vila Real, Portugal; University of Warsaw, POLAND

## Abstract

In a recent paper, we presented new evidence and provided new insights on the status of Cantabrian brown bear subpopulations, relevant for this species conservation. Namely, we revealed the likely phylogeographic relation between eastern Cantabrian subpopulation and the historical Pyrenean population. We have also detected an asymmetric flow of alleles and individuals from the eastern to the western subpopulation, including seven first-generation male migrants. Based on our results and on those of previous studies, we called the attention to the fact that Eastern Cantabrian brown bears might be taking advantage of increased connectivity to avoid higher human pressure and direct persecution in the areas occupied by the eastern Cantabrian subpopulation. In reply, Blanco et al (2020) [11] have criticized our ecological interpretation of the data presented in our paper. Namely, Blanco and co-authors criticize: (1) the use of the exodus concept in the title and discussion of the paper; (2) the apparent contradiction with source-sink theory; (3) the apparent overlooking of historical demographic data on Cantabrian brown bear and the use of the expression of population decline when referring to eastern subpopulation. Rather than contradicting the long and growing body of knowledge on the two brown bear subpopulations, the results presented in our paper allow a new perspective on the causes of the distinct pace of population growth of the two brown bear subpopulations in the last decades. Here, we reply to the criticisms by: clarifying our ecological interpretation of the results; refocusing the discussion on how the new genetic data suggest that currently, the flow of individuals and alleles is stronger westward, and how it may be linked to direct persecution and killing of brown bears. We provide detailed data on brown bear mortality in the Cantabrian Mountains and show that neither migration, gene flow, population increase nor mortality are balanced among the two subpopulations.

## Introduction

Brown bear became extinct in Portugal in the XIX century, with the last report of a bear killing in 1843 [1] but the species persisted in Spain until today in Pyrenean and the Cantabrian mountain ranges [2]. Both populations were at the verge of extinction and are currently recovering [2–5]. The Pyrenean population is nowadays more distinct from historical Iberian populations, because of population reinforcements with brown bears from Slovenia [6], while the Cantabrian population is still free from allochthonous contributions and has been recovering in the last decades [4,5]. The Cantabrian population is structured in two (eastern and western) subpopulations, that were isolated for several decades due to habitat fragmentation, with the consequent reduction on gene flow [2,3,7]. In our paper [8], we found that the mitochondrial lineage present in the eastern Cantabrian subpopulation is closer to the haplotype identified in the historical Pyrenean population [9]. Thus, differentiation among the Cantabrian subpopulations is ancient and cannot be explained, solely, by human-mediated population decline and fragmentation. The non-monophyly of the Cantabrian populations and the relation of the extant eastern Cantabrian haplotype with the historical Pyrenean lineage is supported by the comparison with extinct haplotypes recovered by Valdosiera and co-authors [10]. More recently, gene flow among Cantabrian brown bear subpopulations is increasing, as well as population size [4,5,11,12] even if this increase has been more pronounced in the western Cantabrian subpopulation. We also found solid evidence of migration of males and asymmetrical gene flow from eastern to western Cantabrian subpopulation. We suggested that this “counterintuitive” pattern–given the fact that the western subpopulation is larger and growing at a higher rate–could be explained by a higher level of human disturbance (namely direct persecution) upon the eastern subpopulation. This hypothesis was initially presented by Lamamy and co-authors [13], after finding that habitat alone could not explain the differences in population trends, numbers, and fecundity among the two subpopulations. Blanco and co-authors [11] contested our interpretation of the data, as well as the use of the concept of exodus, the apparent disregard for well-established demographic data and presented anecdotal data on mortality to refute our interpretation.

While acknowledging the misuse of the expression “population decline”, we reply to Blanco et al [11], with the main aim of refocusing the discussion on the most relevant issue, which is to understand the reasons behind the distinct paces of demographic increase in the two subpopulations and the “counterintuitive” migration and gene flow patterns, to inform the Cantabrian brown bear conservation efforts. We also place in context the use of the “exodus” concept and of the reference to source-sink theory in our original paper. Lastly, we provide figures on brown bear mortality in both subpopulations, providing additional support to the role of human persecution as a likely driver of the observed migration patterns.

## Materials and methods

Major criticisms of Blanco et al [11] to our paper [8] are focused on: (1) the use of the concept of exodus applied to asymmetric migration on Cantabrian brown bears; (2) the interpretation of an asymmetric migration itself; (3) an apparent contradiction with classical source-sink theory; (4) and the apparent disregard of long-term and abundant information on the demography of brown bear. We start by re-focusing the use of the concept of exodus (and colonization), as well as the references to source-sink theory by Gregório et al (2020) [8], that are different from the interpretation of Blanco and co-authors [11] Secondly, we present a compilation of data on brown bear mortality going back several decades and discuss these figures at the light of available demographic data on brown bears. In third place, we discuss the potential role of this asymmetric flow of bears from Eastern to Western Cantabrian subpopulations at the light of these mortality data and on previous literature on Cantabrian and other brown bear populations. Last, we attempt to refocus the discussion on–what we consider to be–the most important: the likely role of direct human persecution on the Cantabrian brown bear populations.

## Results and discussion

### Exodus, colonization and source-sink theory

Source-sink theory has been extensively applied in the context of spatially structured populations [14,15] and namely in the case of Cantabrian brown bear population [16]. For a source-sink dynamics to occur in Cantabrian brown bear that would imply an asymmetric migration flow from a (more productive) source to a (less productive) sink population, where mortality would exceed fecundity and the population would survive at the expenses of immigration. A dominant eastward migration pattern has been reported in previous studies [4] and, in terms of Cantabrian brown bear conservation, this would be the most desirable pattern, for it would help to reinforce the most vulnerable (eastern) subpopulation. In the title of our paper [8], we used two concepts for illustrating two alternative outcomes from the use of corridors (and increased connectivity) by brown bears in the Cantabrian mountains; a) either enhanced connectivity would be mainly working as a way for eastward migration, i.e., bears moving mostly from the larger and faster increasing western subpopulation, towards the more vulnerable subpopulation (“colonization”, to illustrate the expected positive effect); or b) it would be mostly used for westward migration of bears, with bears coming mostly from the smaller and more slowly increasing eastern subpopulation (“exodus”, to illustrate an opposite, negative, effect).

While the concept of colonization (also illustrative) is not disputed by Blanco et al [11], the authors question the suitability of the use of the “exodus” concept, for it refers to a mass departure of individuals, and illustrate it with the example of the Exodus of Hebrews from Egypt. We believe that their aim–with the reference to a biblical example, in a scientific publication–was also to illustrate and reinforce their point of view. At some extent, this was our goal with the “intriguing question that headed the title” (page 1) of our paper. However, when using the “exodus” we were not referring to the biblic Exodus, nor specifically to a mass movement of individuals, but to a continued movement of individuals, as in the sense of *rural exodus*, which we feel to be a better comparison in this context. Besides, the use of the exodus concept is not new in the context of ecology and conservation biology. For example, the word has been used for explaining phylogeographic patterns relevant to conservation, as in the case of wildcats [17]. It also has a long history of use in the context of population movements and ecological responses to human disturbance [18–20] and it has even been used in the specific context of connectivity and ecological corridors [21]. Therefore, we fail to see how the opinion of the authors about the use of the “exodus” concept could be used as a line of evidence to dispute or criticize the interpretation of results in our paper [8], mostly because the word is used as an interrogation and not as a statement in the title of the original paper.

Blanco and co-authors [11] refer that “in ecology, the dynamics of spatially structured populations (like that of Cantabrian brown bear) is usually explained in the framework of the source-sink theory” (page 3). However, the concept is not adequate to explain the dynamics of all structured populations, but of those (sink) wherevreproduction is insufficient to balance mortality, and the population is maintained by continued immigration from more-productive source populations [14,15]. While a diversity of source and sink populations have been defined, the dependency of immigration to counterbalance mortality is part of the definition of sink populations. We do not question the importance of source-sink theory, namely in the context of metapopulations, but whether if the concept applies to the Cantabrian brown bear context. As Blanco and co-authors [11] refer, the concept of attractive sink [22] has been suggested for the case of Cantabrian brown bear subpopulations [16]. However, the authors of this study identified the western subpopulation–that was characterized as having good quality habitat and high human impact–as the attractive sink, and not the eastern subpopulation. Additionally, Naves and co-authors [16] did not use any information on direct persecution (namely hunting or poaching) for characterizing human impact. We are aware of the reasons why it is difficult to measure mortality or to assess hunting or poaching, for bear hunting is not legal in Spain and poaching does not occur in plain sight.

Nevertheless, poaching has been often mentioned as a major challenge to the conservation of the Cantabrian brown bear and other threatened European populations [23,24] and the assumption that more people (namely higher population density or more tourists) are adequate proxies for the level of disturbance in brown bear is arguable. In fact, it is reasonable to consider that in areas where local populations benefit from tourism (namely for brown bear observation), attitudes toward bears could be more positive, and mortality eventually lower. Moreover, Lamamy et al [13] have recently shown that habitat differences among subpopulations were not enough to explain demographic differences and pointed the “persistence of poaching and/or bad hunting practices during hunting” as a major driver of differences among both subpopulations.

Blanco and co-authors [11] refer to several examples in the literature to question the possibility “of mass dispersal of large carnivores from areas with high mortality to areas with low mortality”. Again, they provide data on human density and tourism in the two Cantabrian subpopulations to illustrate how bears would respond to human disturbance, by changing their activity patterns rather than performing long distance migrations. Blanco et al (2020) [11] refer to data on mortality, compiled by the authors, but these data are not presented. Moreover, the examples provided by Blanco and collaborators refer to very different contexts, in North America and North Europe, where brown bear inhabits large wild areas, legal hunting does occur, and human densities are lower. In more similar contexts, such as other threatened brown bear populations in southern Europe, illegal killing or poaching are often pointed as a major threat [2,24,25]. We will return to this question and to the reasons that led us to suggest that brown bears might be migrating westward to avoid human persecution, after we present data on brown bear mortality.

### Cantabrian brown bear mortality in the last decades

Blanco and co-authors [11] rejected our interpretation by stating, among other reasons, that there was no evidence of higher mortality of brown bears in the Eastern subpopulation. Based on the mortality data compiled by FAPAS (whose members co-author this paper) during the last decades, we provide here a summary (Table 1) on brown bear mortality by subpopulation, period and sex. More detailed information is provided in Supplementary information (S1 Table). Similar patterns can be found identified (S2 Table) using mortality data collected by Palomero and co-authors [26]. In the data compiled by these authors, it is important to notice that: (i) the sex of the bear and the cause of death are much more often unknown in the eastern population (S2 Table); (ii) when excluding deaths with unknown cause, the proportion of deaths caused by poisoning, shooting or snaring, relatively to natural causes, is much higher in the eastern (7 criminal to 2 natural) than in the western subpopulation (8 criminal to 11 natural); (iii) mortality in proportion to current population size is four times higher in the eastern subpopulation.

**Table 1:** Reported brown bear deaths (and as percent of current population size) in the two Cantabrian (eastern and western) populations, from 1977 to 2020. Population size estimates refer to estimates provided by Palomero and co-authors [26]. Numbers of deaths, compiled by the authors of this paper, are presented by period and sex. Details on each death report are provided in supplementary information (S1 Table).

	Population Size (2019–2021)	Reported deaths (until 1999)	Reported deaths (2000–2010)	Reported deaths (since 2011)
***Eastern Subpopulation***	50	7 (14%)	7 (14%)	13 (26%)
**Males**		5	2	2
**Females**		1	1	2
**Unknown**		1	4	9
***Western Subpopulation***	280	10 (4%)	8 (3%)	19 (7%)
**Males**		1	4	12
**Females**		5	0	4
**Unknown**		4	4	3

Blanco et al [11] have also criticize us for disregarding demographic data on Cantabrian brown bear. We had into account demographic data and used the data compiled by Caussimont & Hartasanchez [5], which–at the time–summarized the most updated data, namely the results from the 2015 Census coordinated by the Autonomous Provinces of Spain, as well as the data presented by Perez et al [12] and also used by Penteriani and co-authors [27]. We must hold in mind that, when we refer to a likely higher mortality in the eastern subpopulation, we cannot (and were not) referring to absolute numbers, because mortality must always be interpreted in relation to population size. While assuming that both subpopulations have continued to increase, comparable official data were, at the time, not available for the two subpopulations since 2015. More updated numbers are currently available [26] and we included it here. The figures we provide (Table 1) show that mortality is far from being balanced and–considering the last years and ignoring the cases where sex is unknown–a more balanced mortality of females and males is found in the Eastern subpopulation. Numbers from western subpopulation suggest that mortality is currently more intense over males, here. Particularly if we consider the lower (though increasing) number of reproductive females provided by Blanco et al [11], female mortality might have dramatic effects particularly in the Eastern subpopulation. When we discussed the impact of habitat, sex ratio or direct persecution [8], we did not present those as mutually exclusive, and admit that direct persecution might have a direct (as a factor of disturbance) or indirect (through the effect over females and sex ratio) in the subpopulation. Also, besides the direct effect on mortality, direct persecution (poaching) or bad hunting practices may affect populations differently. While we do not neglect the abundant literature referred by Blanco et al [11] on compensatory immigration from low to high-mortality areas, we cannot also neglect the migration patterns, identified in our paper [8] nor the mortality numbers or the link to brown bear persecution presented here.

### Is population growth comparable in the two subpopulations?

We accept one of the criticisms made by Blanco et al [11] on our paper. We did use the expression “population decline” in Gregório et al [8], which is not accurate, particularly in regard of the absolute population increase in both populations. We should have referred to the slower pace of population increase in the Eastern subpopulation, which would have been a more accurate characterization of the situation. Nevertheless, there is still an unbalance among the population trend or the number of females with cubs of the year (FCOY) among the two subpopulations, even if these numbers are analyzed in reference to subpopulation size.

In first place, we would like to give a word of caution about the methodology that is used for estimating the number of reproductive females based on the FCOY data of each biennium. The sum of the FCOY numbers for every two years is based on brown bear biology and the assumption that each female reproduces every two year and therefore the population of reproductive females is only complete if we take into account two reproductive years. While considering that this is a correct approach in relatively undisturbed populations, we believe this practice might be overestimating–for both subpopulations–the numbers of reproductive females because it does not account for the chance of cub mortality. In brown bear populations highly disturbed by hunting or poaching, there is a risk of cub mortality or lowered recruitment [23,28,29] and, if a female loses her cubs of the year, she would be likely to reproduce again in the following year and would be counted twice. We consider that there is room for reasonable doubt in the context of Cantabrian brown bear subpopulations, for there is evidence of long-term and ongoing disturbance and direct persecution in these two brown bear subpopulations.

Nevertheless, by applying the same reasoning to both subpopulations and using numbers from the same periods, the two subpopulations are increasing at different paces. In what concerns to the FCOY numbers per year, and taking the numbers provided by Palomero et al [26], we have the following scenario for 2018: 7 FCOY in the eastern subpopulation and 31 in the western subpopulation. The 2018 figures are consistent with the average number for the 2007–2018 period: 4.5 FCOY in the eastern subpopulation vs 26.3 in the western subpopulation. Comparable numbers for the period 1994–2004 can be estimated from the figures provided by Palomero and co-authors [30]: average FCOY of 1.27 for eastern subpopulation and 7.27 for western subpopulation. Thus, while the FCOY increased by 3.3 times in the eastern subpopulation, between the two periods, in the western subpopulation it increased by 4 times. Thus, considering that the eastern subpopulation departed from a much lower figure, the increase in FCOYwas similar on both subpopulations.

On the other hand, from the early 90’s to 2018, the western Cantabrian subpopulation has increased by four times in size (from 65 to 280 individuals) while the eastern Cantabrian subpopulation only doubled in the same period (20 to 50 individuals; ratio 2.5), based in the numbers estimated by Clevenger & Purroy [31], Naves and Palomero [32] or, more recently, the brown bear census held by the Spanish Autonomic Communities, together with several NGOs, in 2015, and Palomero et al [26]. Therefore, even with a similar increase in fecundity, apparently, that did not result in similar population increase. The reasons why higher fecundity does not translate in comparable population growth should be the focus of this discussion.

### Is migration balanced among the two subpopulations?

In their reply, Blanco et al provide an historical summary on the reports of migration among the two subpopulations. The report is overall accurate, and we will only focus on where we consider to be some inconsistencies. We are aware of previous studies–which we do refer in our paper [8]–namely the one by Pérez and co-authors [3], reporting bidirectional male migration, though mainly from east to west, based on samples collected mainly between 2004 and 2007. The authors refer to a more recent account by Gonzalez et al [4], based on samples collected in 2013–2014, suggesting higher eastward migration and admixture. However, as the authors acknowledge, sample sizes were unbalanced and assignment of migrants and admixed individuals was not based on any formal testing, so results should be viewed with caution. Moreover, only information on microsatellite genotypes was used to infer migration and admixture. In our study, to account for unbalanced sample size, we performed analysis using rarefied samples and formally tested for migrants and admixed individuals. Additionally, in Gregório et al [8], information for asymmetric flow came from microsatellite genotypes (which are “re-shuffled” every generation and thus are more adequate for short-term processes), alleles (which tend to hold information of immigration for a longer period) and a mitochondrial gene (which, mostly in a species with female philopatry, allow for the assessment of ancestry). In all cases, gene flow or migration was asymmetric, and mainly eastward.

Blanco et al [11] reinterpret our results in order to show higher admixture in the eastern subpopulation. They do it in terms of “numbers of admixed individuals”. Because sample sizes are unbalanced, we consider that analysis based on population gene pools (actually, Bayesian assignment algorithms work on a basis of optimization of population, rather than individual parameters) are less prone to error and thus report proportions per subpopulation as well. In that sense, admixture in the eastern subpopulation is–at most–equivalent to admixture in the western subpopulation, if migrants are excluded from the analysis. Blanco et al [11] also suggest interpreting the data by pooling results from the several studies together. However, and since demographic processes are dynamic, shifts in dominant migration flow are likely to occur. Most updated evidence suggests it is currently stronger towards west.

Last, a word on the assumption by Blanco et al [11] that our use of the word exodus would necessarily imply balanced male and female migration. That is not true even for human populations, as can be observed in many contemporary migration waves, where man often migrate first in order to find suitable conditions for their families. It is also arguable that balanced migration should be expected for species with female philopatry, as the brown bear. With the use of the concepts of exodus and colonization, we did not intend to coin a new ecological theory but illustrate two different outcomes of increased connectivity.

## Conclusions

Cantabrian brown bear subpopulations have been recovering from the verge of extinction during the last decades. Successfull recovery of these subpopulations would not been possible without the contribution of several governmental and non-governamental organizations, as well as numerous people living and working in “brown bear territories”. However there are still differences between the two subpopulations. We accept one of the criticicisms by Blanco et al [11]–the lack of accuracy when referring to population decline in the eastern subpopulation–but this is the only criticism based on factual information. It is true that none of the two subpopulations is currently declining, but the situation is different in the two subpopulations. While fecundity appears to have increased at similar rates in both subpopulations, the eastern subpopulation is still growing at a much slower rate, receiving less migrants and gene flow and showing higher mortality. The focus of the discussion should be on identifying the reasons behind these differences. The new evidence we brought [8], together with the mortality data presented here, and elsewhere [26], help understand the differences and act upon the causes. Thus, the best efforts of all are necessary for the successful conservation of the Cantabrian brown bear. In this regard, a focus on human-bear conflict is paramount, not only to assure survival of bear in areas where the species is currently present, but also in areas of bear expansion, as in Galiza and North Portugal.

## Supporting information

S1 Table Mortality data compiled by the authors. Details on the brown bear deaths reported in the Cantabrian populations, from 1977 to 2020.
(XLSX)

S2 Table Mortality summary based on Palomero et al (2021) [21]. Summary on mortality based on the data compiled by Palomero et al (2021) [21]. Data are available in Table 2, page 16 of the referenced publication.
(XLSX)
